# Exploration of the feasibility of clinical application of phage treatment for multidrug-resistant *Serratia marcescens*-induced pulmonary infection

**DOI:** 10.1080/22221751.2025.2451048

**Published:** 2025-01-07

**Authors:** Xiangke Duan, Wenfeng Liu, Yanyu Xiao, Man Rao, Liyin Ji, Xiaofu Wan, Shuhong Han, Zixun Lin, Haichen Liu, Peifen Chen, Kun Qiao, Mingbin Zheng, Jiayin Shen, Yang Zhou, Tetsuya Asakawa, Minfeng Xiao, Hongzhou Lu

**Affiliations:** a Department of Infectious Diseases, National Clinical Research Center for Infectious Diseases, Shenzhen Third People’s Hospital, Shenzhen, People’s Republic of China; b BGI Research, Shenzhen, People’s Republic of China; c Department of Clinical Laboratory, National Clinical Research Center for Infectious Diseases, Shenzhen Third People’s Hospital, Shenzhen, People’s Republic of China; d Department of Infection and Immunology, National Clinical Research Center for Infectious Diseases, Shenzhen Third People’s Hospital, Shenzhen, People’s Republic of China; e School of Medicine, Southern University of Science and Technology, Shenzhen, People’s Republic of China; f Department of Respiratory Medicine, National Clinical Research Center for Infectious Diseases, Shenzhen Third People’s Hospital, Shenzhen, People’s Republic of China; g Department of Thoracic Surgery, National Clinical Research Center for Infectious Diseases, Shenzhen Third People’s Hospital, Shenzhen, People’s Republic of China; h Department of Science and Education, National Clinical Research Center for Infectious Diseases, Shenzhen Third People’s Hospital, Shenzhen, People’s Republic of China; i Institute of Neurology, National Clinical Research Center for Infectious Diseases, Shenzhen Third People’s Hospital, Shenzhen, People’s Republic of China

**Keywords:** Phage treatment, phage Spe5P4, *Serratia marcescens* (*S. marcescens*), multidrug-resistance, efficacy and safety

## Abstract

*Serratia marcescens* (*S. marcescens*) commonly induces refractory infection due to its multidrug-resistant nature. To date, there have been no reports on the application of phage treatment for *S. marcescens* infection. This study was conducted to explore the feasibility of phage application in treating refractory *S. marcescens* infection by collaborating with a 59-year-old male patient with a pulmonary infection of multidrug-resistant *S. marcescens.* Our experiments included three domains: *i*) selection of the appropriate phage, *ii*) verification of the efficacy and safety of the selected phage, *iii*) confirmation of phage-bacteria interactions. Our results showed that phage Spe5P4 is appropriate for *S. marcescens* infection. Treatment with phage Spe5P4 showed good efficacy, manifested as amelioration of symptoms, hydrothorax examinations, and chest computed tomography findings. Phage treatment did not worsen hepatic and renal function, immunity-related indices, or indices of routine blood examination. It did not induce or deteriorate drug resistance of the involved antibiotics. Importantly, no adverse events were reported during the treatment or follow-up periods. Thus, phage treatment showed satisfactory safety. Finally, we found that phage treatment did not increase the bacterial load, cytotoxicity, virulence, or phage resistance of *S. marcescens,* indicating satisfactory phage-bacteria interactions between Spe5P4 and *S. marcescens*, which are useful for the future application of phage Spe5P4 against *S. marcescens.* This work provides evidence and a working basis for further application of phage Spe5P4 in treating refractory *S. marcescens* infections. We also provided a methodological basis for investigating clinical application of phage treatment against multidrug-resistant bacterial infections in the future.

## Introduction

*Serratia marcescens* (*S. marcescens*) is a ubiquitous gram-negative opportunistic pathogen [1], which can infect a wide range of hosts, including insects, plants, mammals, and humans [2]. *S. marcescens* is an opportunistic pathogen that commonly occurs in immunocompromised individuals, such as neonates and patients in intensive care units (ICU). *S. marcescens* may cause systemic infections, particularly those in the respiratory system, urinary tract, wounds, and bloodstream [3]. *S. marcescens* infections are usually considered a serious problem in the clinical setting because *S. marcescens* is intrinsically resistant to a spectrum of antibiotics, such as β-lactams, aminoglycosides, fluoroquinolones, macrolides, and cationic antimicrobial peptides. Moreover, some emerging multidrug-resistant strains may pose additional challenges for clinical management due to their carriage of resistance determinants, such as extended-spectrum β-lactamases and MCR (mobile colistin resistance) enzymes [1]. Guo’s study further contributes to this understanding by revealing that a high percentage of Aeromonas strains – specifically, 96% (1,168/1,217) – carry at least one MCR family protein, thereby advancing our knowledge of colistin resistance [4]. Accordingly, in addition to the development of novel antibiotics, many novel therapeutics have been developed, including phage treatment.

Phages represent a diverse group of viruses that are ubiquitous in nature, exhibiting remarkable specificity toward bacterial hosts [5]. Phages can be considered as potential therapeutic agents because of their capacity to infect and lyse specific “harmful” bacterial strains, whereas leaving “beneficial” microbiota unaffected. The exquisite nature of phages against their target bacteria allows us to consider them as a precision-guided approach to treat certain bacterial infections [6]. Phages have been used to treat various infections caused by drug-resistant bacteria [7], including surgical wounds, respiratory tract infections [8], and urinary tract infections [9]. The phages used in treatment should exhibit key characteristics, such as strong lytic activity, a broad host range, or good tolerance. Examples of such phages have been successfully applied to a range of challenging pathogens, including *Acinetobacter baumannii* [10], *Escherichia coli* [11, 12], *Pseudomonas aeruginosa* [13–15], *Klebsiella pneumoniae* [16, 17], *Mycobacteria* [18, 19], *Staphylococcus aureus* [20, 21], *Salmonella* [22–25], *Cronobacter sakazakii* [26], and *Aeromonas hydrophila* [27]. Nevertheless, to our knowledge, there is no report yet on the application of phage treatment for *S. marcescens* infection*,* which is challenging in clinical practice.

To develop a phage-based therapy against *S. marcescens,* several issues must be considered: *i)* selection of an appropriate phage; *ii)* verification of the safety and efficacy of this phage when combined with antibiotics; *iii)* elucidation of the nature of phage-bacteria interactions, which is important for further usage. A section of a proper patient for treatment is also vital for the feasibility of the entire protocol. Fortunately, we admitted a patient with a refractory pulmonary infection with *S. marcescens,* which allowed us to design and implement the present study. In collaboration with this patient, we conducted this study and attempted to answer the following three questions: *i)* Which phage is appropriate for the treatment of multidrug-resistant *S. marcescens* infections? *ii)* What about its safety and efficacy, as well as its synergistic effects with antibiotics? *iii)* What are the phage-bacteria interactions? We believe that the findings of the present study may provide useful insights and experiences to develop phage-based treatment against refractory *S. marcescens* infection, which still lacks a specific treatment.

## Materials and methods

### Experimental design

This study was designed with four contents: (1) Selection of a patient with a refractory infection with multidrug-resistant *S. marcescens*. (2) Selection of an appropriate phage with characteristics of safety, free of virulence, lysogeny, and antibiotic-resistance genes, and with satisfactory synergic effects when combined with antibiotics. (3) Verification of the efficacy and safety of phage treatment. (4) Confirmation of phage-bacteria interactions.

#### Patient

One patient with refractory pulmonary infection was recruited for this study. This study was conducted in strict compliance with the guidelines of the Declaration of Helsinki of the World Medical Association (2013) and approved by the ethical committee of the Third People's Hospital of Shenzhen (approval no: 2021-068-03). This patient provided signed informed consent when he was introduced in detail to all the contents concerning the novel phage treatment and the research protocol by the medical staff. He agreed to publish his case in a scientific article and his personal information was strictly protected according to the Personal Information Protection Law of the People's Republic of China.

#### Clinical setting

**Figure 1.**
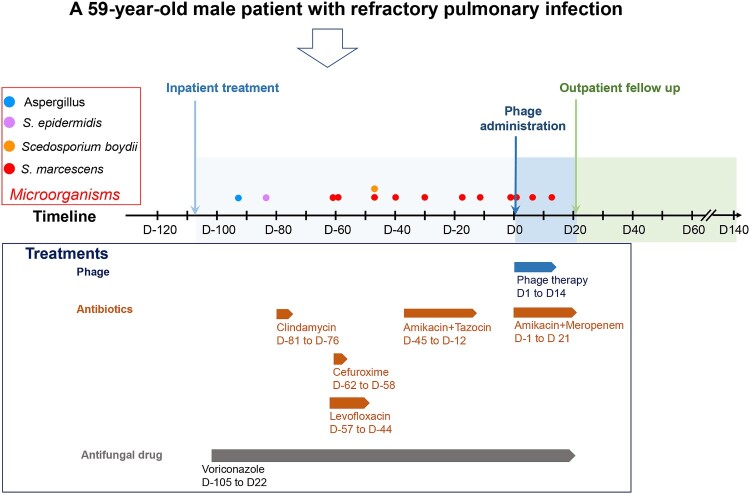
**Protocol of the treatments and examination of the microorganisms associated with phage treatment.** This diagram presents the treatments and examination of microorganisms over time in this patient. Results of microorganism examinations are shown as different colours. This patient underwent three types of treatment: 1. Antifungal treatment: Voriconazole (150 mg, q12 h); 2. Antibiotics treatments: Clindamycin (0.6 g, q12 h), Cefuroxime (1.5 g, q12 h), Levofloxacin (0.5 g, q.d.), Amikacin/Tazocin (0.8 g/4.5 g, q.d./q8 h), Amikacin/Meropenem (0.4 g/1 g, q8 h); 3. Phage therapy (10^9^ PFU/mL, q12 h).

**Figure 2.**
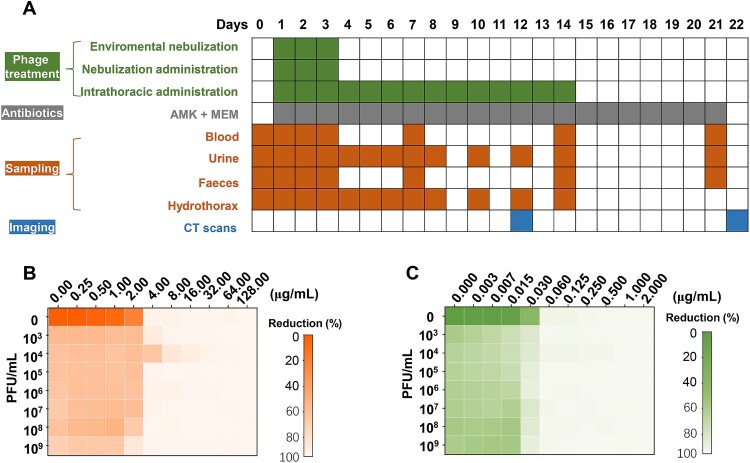
**Integrated phage treatment of *S. marcescens* infection and the related synergic effects.** (A) Protocol of treatments and examinations. (B) Verification of the synergic effects of phage Spe5P4 + AMK. (C) Verification of the synergic effects of phage Spe5P4 + MEM. AMK: amikacin; MEM: meropenem.

The entire clinical protocol is shown in the Figure 1, which was adjusted according to the actual clinical response to the treatments. The entire follow-up period was 260 days, including 120 days before phage treatment (recorded as “D-120”) to 140 days after phage treatment (recorded as “D140”). The patient underwent three types of treatment: antifungal, antibiotic, and phage treatments. The detailed medication strategy is shown in Figure 1. The presence of microorganisms in clinical samples was also examined (Figure 1). Four types of clinical samples, namely, blood, urine, feces, and hydrothorax, were collected (Figure 2A). Sampling of blood, urine, and feces was performed in accordance with clinical routine. The hydrothorax was corrected by standard real-time transthoracic ultrasound (TUS) – guided thoracentesis, as described in a previous study [28]. In brief, all thoracic surgeries were performed by an experienced chest physician. Guided by an ultrasound scanner, especially for TUS examination (Resona7, Mindary, Shenzhen, China), a 5 MHz probe with a holed guide was used to determine the insertion point of thoracentesis. TUS was used to exclude possible pneumothorax before the procedure. The patient sat on a bed with his arms placed on a table. The patient’s position was adjusted to ensure that the hydrothorax was clearly visible on B-mode TUS. The needle insertion point was selected below the top of the effusion and the ribs were shunned. sterilization with 1% povidone-iodine and anesthesia with 2% lidocaine, a 20 G needle with a 50 mL syringe was inserted into the intercostal space and approached to the effusion. Approximately 5 mL of the fluid was collected for examination. The patient did not complain of any discomfort during the thoraces. The detailed sampling schedule is shown in Figure 2A. Chest computed tomography (CT) scans with thin sections (0.65-2 mm) were performed during the entire study. The detailed parameters of the CT scans/machines were similar to those used in our previous study [29].

### Selection of the appropriate phage

#### Bacterial culture and antibiotic susceptibility tests

*S. marcescens* strains were grown at 37˚C in lysogeny broth (LB) medium or on solid LB agar plates (LBA) supplemented with 1.0% (w/v) agar. Bacterial growth was measured using a spectrophotometer (Ultrospec 10, Biochrom, Massachusetts, US) and expressed as optical density at 600 nm (OD_600_). For the phenotype assays, *S. marcescens* strains were propagated from an overnight culture and subsequently inoculated at a 1:50 dilution in fresh LB medium. The inoculum was incubated at 37°C while agitating at 200 rpm for 3 h, and the OD_600_ of 1.0 for further assays.

Two sets of standard antibiotic susceptibility tests (before and after phage treatment) introduced in a previous study [30] were performed using hydrothorax samples. Briefly, bacteria isolated from hydrothorax samples were identified using matrix-assisted laser desorption ionization-time of flight mass spectrometry (MALDI-TOF MS, Microflex LT/SH, BRUKER, Massachusetts, US). All isolates were labelled and preserved in 20% (v/v) glycerol at −80°C. Antibiotic susceptibility of *S. marcescens* isolates was ascertained using the Phoenix M50 system (Phoenix M50, bioMéreux, Marcy-l'Étoile, France). Tests determining the minimum inhibitory concentrations (MICs) of antibiotics, including ampicillin, amoxicillin, gentamicin, tobramycin, amikacin (AMK), chloramphenicol, tetracycline, cefoxitin, cefazolin, cefuroxime, ceftriaxone, levofloxacin, ciprofloxacin, imipenem, meropenem (MEM), ertapenem, and polymyxin B, were conducted according to the Performance Standards for Antimicrobial Susceptibility Testing issued by the Clinical Laboratory Standards Institute (CLSI) [31]. Categorization of MICs as well as determination of “susceptible,” “resistant,” and “intermediate” were performed as per the M100 Performance Standards for Antimicrobial Susceptibility Testing introduced in the CLSI guidelines [31].

#### Phage isolation, propagation and characterization

Phages were isolated and purified using the enrichment method [32] with *S. marcescens* 328505 as the host (D-62). Briefly, the sewage water collected in Longgang District, Shenzhen, was filtered through 0.22 μM syringe filter and mixed with 5 mL 2×LB, and 100 μL exponential phase culture of *S. marcescens* 328505 was added. The mixture was incubated overnight at 37°C, with agitation at 200 rpm. After incubation, the mixture was centrifuged at 10,000×g for 5 min and subsequently filtered using a 0.22 μM syringe filter. The resulting phage lysate was purified using five consecutive single-plaque isolations and propagation. Phage titre was quantified as the number of plaque-forming units (PFU/mL) using the double agar overlay plaque assay method. The morphological characteristics were observed using an electron microscope (JEM1200EX, JEOL, Beijing, China). The phages were transferred onto carbon-coated copper grids for 30 s to settle the particles. They were then stained with 1% of uranyl acetate for 40 min. The grids were examined at 250,000 × magniﬁcation using an electron microscope (JEM1200EX, JEOL, Beijing, China). To confirm the other characteristics of the phage, the temperature and pH stabilities, as well as the optimal multiplicity of infection (MOI) of the phage, were also tested. To assess the pH stability, phages were cultured at a concentration of 5 × 10^9^ PFU/mL in LB with pH levels ranging from 2.0–12.0 at 37°C for 1 h. The proportion of persistent phage particles that retained infectivity against the host strain was evaluated using the double-agar overlay method. To investigate the effect of temperature, phages were cultured at a concentration of 5 × 10^9^ PFU/mL in LB at temperatures ranging from 4–80°C for 1 h. Following incubation, the viability of the phage particles was measured using the double agar overlay method on the host strain. To determine the optimal multiplicity of infection (MOI), *S. marcescens* was adjusted to a concentration of 1 × 10^8^ CFU/mL. The phage solution was then mixed with diluted bacteria at ratios of 0.00001, 0.0001, 0.001, 0.01, 0.1, and 1. The mixture was incubated for 3 h at 37°C. Subsequently, the culture mixture was centrifuged at 12,000 rpm for 5 min, and the supernatant was filtered through a 0.22 μm filter. The titre of the phage filtrate was determined using the double layer plate method.

#### Genome assembly, annotation, and comparison

The raw data were filtered using Trimmomatic v0.39 [33] to remove reads with more than 30% match to the adaptor sequence, and at the same time, reads with an average quality of less than 20 bases were removed. Validate the filtered data with FastQC v0.11.9, and calculate the read count with readfq. Kraken 2 was used for species annotation of the sequencing data. SPAdes v1.1.0 [34] was used for the de novo genome assembly and assembly drafts. The quality of the assembled sequences was checked using Quast v5.0.2, contigs shorter than the specified length (default 500 bp) and alignments shorter than the specified length (default 65 bp) were removed. The size, number of contigs, and GC content of assembled sequences were determined. Finally, CheckM was used to obtain the completeness and contamination rate of the assembly sequences, and sequences with less than 95% completeness and more than 1% contamination were excluded. Prokka v1.14 [35] was used to annotate the phage genome, and Proksee was used to visualize the phage genomes [36]. A phylogenetic tree was constructed using the PhageScope database (https://phagescope.deepomics.org/) [37] and iToL v6 (https://itol.embl.de/) was used for modification and presentation [38].

#### Genome DNA extraction and sequencing

The purified phage was treated with DNase I (PROMEGA, Madison, US) and 6 U/mL and RNase (Takara, Shiga, Japan) at 50 μg/mL for 1 h at 37°C. Next, 20 μL of 2 M ZnCl_2_ was added and incubated for 5 min at 37°C. Following centrifugation at 10,000 × g for 1 min, the supernatant was discarded and the pellet was suspended in 500 μL TES buffer. Protein digestion with Proteinase K (TIANGEN, Beijing, China) (20 μL) was performed at 37°C for 90 min. Genomic DNA precipitation involved the addition of 60 μL of 3M potassium acetate, followed by a 15-minute incubation on ice. Subsequent protein extraction steps were conducted using a mixture of phenol:chloroform:isoamyl alcohol (25:24:1, v/v) and chloroform: isoamyl alcohol (24:1), repeated twice. Genomic DNA was precipitated with isopropanol for 30 min, washed twice with 70% ethanol, centrifuged at 10,000 g for 5 min. The resulting genomic DNA was dissolved in 20 μL sterile water and stored at – 20°C. Genomic DNA was sequenced by Novogene Co. Ltd. (Beijing, China).

After confirming the results, we selected phage Spe5P4 for subsequent experiments.

#### Phage resistant *S. marcescens* induction assay

Exponential phage culture of *S. marcescens* 328505 was adjusted to 1 × 10^6^CFU/mL and co-cultured with Spe5P4 for 24 h at MOI of 100, 10, 1, 0.1, 0.01, 30 replicates for each MOI. One colony was chosen randomly from each plate and colony-puriﬁed three times [39]. Those that persistently showed no plaques were confirmed as phage-resistant strains.

#### Confirmation of the synergic effects combining phage Spe5P4 and antibiotics

Because the efficacy of phage Spe5P4 was uncertain at the start of the study, we evaluated the efficacy and safety of phage + antibiotics rather than the sole administration of phage to the patient. AMK and MEM were selected for the combined treatment with phage Spe5P4 because of their low cytotoxicity and efficacy against biofilms. Phages were administered in three ways: Environmental nebulization: 3 mL 10^9^ PFU/mL phage mixed with 2 mL 0.9% NaCl. Nebulization administration: 0.5 mL 10^9^ PFU/mL phage mixed with 4.5 mL 0.9% NaCl and administered through a nebulizer. Intrathoracic administration: 0.5 mL 10^9^ PFU/mL phage mixed with 4.5 mL 0.9% NaCl and administered through thoracic injection. Antibiotics Amikacin/Meropenem (0.4 g/1 g, q8 h) were administered intravenously. The treatment protocol is shown in Figure 2A. To assess the synergistic effect between phages and these antibiotics, bacterial cultures were exposed to varying concentrations of antibiotics (0.25 μg/mL to 128 μg/mL for AMK, 0.003 μg/mL to 2 μg/mL for MEM with two-fold increasing) spanning multiple magnitudes above the MIC in conjunction with different phage titres (10^3^ PFU/mL to 10^9^ PFU/mL). This assessment was performed using an optically-based microtiter plate assay system (Synergy H2, Agilent Bio Tek, Massachusetts, US), with bacterial growth monitored by OD_600_ at 37℃ over a 24-hour period. The absorbance data were then translated into a heat map to display the percentage reduction in the bacterial population.

Using these methods, phage5P4 was ultimately selected as the appropriate phage for subsequent treatments.

#### Phage preparation

*S. marcescens* 328505 strain (1 mL) was grown in LB medium 100 mL overnight and was incubated in exponential phase, where 1 mL of phage Spe5P4 was added and incubated for 6 h at 37℃. The culture was then transferred to a 50 mL Falcon tube and centrifuged at 10,000 × g for 10 min, followed by filtration sterilization (0.22 μm) of the supernatant. Triton X-100 was added to achieve a final concentration of 5% and the mixture was centrifuged at 10,000 × g for 10 min after incubation at 37°C and 220 rpm. The supernatant was then dialyzed using Biotech CE Tubing (MWCO 1000 kDa, Repligen, Massachusetts, USA) in the 0.9% NaCl to eliminate Triton X-100. The preparation was further concentrated using PEG8000, followed by extraction with chloroform. Subsequently, dialysis was performed using Biotech CE Tubing in 0.9% NaCl to remove residual chloroform. The phage preparation was then sterilized through 0.22 µm filters. The titre of phage preparation was 2.7 × 10^10^ PFU/mL, and the endotoxin levels were evaluated with an End-Point Chromogenic Endotoxin Test Kit (Bioendo, Xiamen, China), and the endotoxin level was 60.3 EU/mL. Finally, the phage preparations were stored at 4℃ for the following experiments.

### Verification of the efficacy and safety of the phage Spe5P4 treatment

The efficacy of phage treatment was confirmed by the amelioration of symptoms, along with chest CT scans and examinations of the hydrothorax. To evaluate the safety of the phage, routine laboratory examinations, including hepatic function (aminotransferase (AST), alanine aminotransferase (ALT)) and renal functions (urea and creatinine (CR)), immunity-related indices (interleukin-6 (IL-6), IL-8, C-reactive protein (CRP), and neutrophil percentage (NEUT%)), and indices of routine blood examination (white blood cell (WBC) and erythrocyte sedimentation rate (ESR)) were checked for association with phage administration. The timing of the examinations is shown in Figure 4. Moreover, a second antibiotic susceptibility test was performed to confirm whether phage Spe5P4 treatment could influence the susceptibility to these antibiotics.

### Microbiological monitoring during phage therapy: confirmation of the phage-bacteria interactions.

#### Lactate dehydrogenase (LDH) assay

Cytotoxicity of the isolated strains was assayed using the A549 cell infection assay. A549 cells (purchased from American Type Culture Collection) were grown in 48-well plates in Dulbecco’s modified Eagle’s medium (DMEM), GlutaMAX, sodium pyruvate, and phenol red supplemented with 10% FBS. Prior to infection, the conﬂuent A549 cells were washed twice with sterile PBS and incubated in DMEM. Log-phase cultures were washed twice with sterile PBS and resuspended in DMEM without FBS. A549 cells were infected with bacteria at a MOI of 10 at 37 ℃ in 5% CO_2_ incubator. Six timings of *S. marcescens* isolates (D0, D7, D8, D12, D14, and D15) were evaluated. After 4 h of infection, the culture supernatants were collected to detect LDH activity at an MOI of 10, which were detected using a commercially available LDH cytotoxicity kit (YAESEN Bio, Shanghai, China) according to standard procedures.

#### Galleria mellonella larvae infection assay

The virulence of *S. marcescens* was evaluated using a *G. mellonella* infection model [40]. The larvae were stored at 4°C and used within a week. A modified infection technique developed by Han *et al*. [41] was used for *G. mellonella* infection. Larvae were inoculated via injection of 500 colony-forming units (CFU) of *S. marcescens* for each isolate in a 20 μL volume into the hind leg using a 30 G needle. Six timings of S. marcescens isolates (D0, D7, D8, D12, D14, and D15) were evaluated. After inoculation, the infected larvae were maintained at 30°C. Each experimental group consisted of 20 larvae per treatment, with the negative control group injected with PBS. *G. mellonella* were observed hourly starting 12 h post-infection and recorded as dead when they did not move in response to touch.

#### Verification of the phenotypic heterogeneity of *S. marcescens* isolates

During phage Spe5P4 treatment, five *S. marcescens* strains were isolated on D7, D8, D12, D14, and D15. To assess the potential changes in phenotypic traits during phage Spe5P4 treatment, we further characterized the isolates in terms of biofilm formation and motility capacity. Microtiter crystal violet (CV) biofilm assays were performed as described previously [42]. Briefly, an exponential culture with an OD_600_ of 1.0 was diluted in fresh LB medium at a ratio of 1:100. Aliquots (100 μL) of diluted cultures were transferred into a 96-well round-bottom plate (Corning) and incubated for 16 h at 37 ℃. Biofilms formed by *S. marcescens* strains were stained with 0.1% CV, and the stained CV was dissolved in 30% acetic acid to measure of the absorbance using a microtiter reader (Synergy H2, Agilent Bio tek, Massachusetts, US, λ = 550 nm). Motility assays, including the capacity for swarming and swimming, were also conducted. For swarm motility, 2 μL of the bacterial suspension was spotted onto 0.7% LB agar plates. For the swimming motility, 2 μL of the bacterial suspension was deposited onto 0.3% LB agar plates. These plates were then incubated at 28°C for 16 h to allow for the growth and observation of motility characteristics using an imaging system (CZONE 6, Shineso, Hangzhou, China).

### Statistics

Statistical analysis was conducted using GraphPad Prism 9.0 software (V9.5.1, GraphPad Software, MA, US). The normal distribution of the experimental data was tested using the Kolmogorov–Smirnov test. The data were then analyzed using t-tests and one-way analysis of variance (ANOVA) with Tukey post-hoc correction. *p* < 0.05 was considered as significantly different.

## Results

### Case presentation

A 59-year old male patient who complained of persistent chest discomfort, shortness of breath, and intermittent cough accompanied by sputum production was recruited. His symptoms persisted until June 2023. Chest CT imaging revealed left-sided pneumothorax; therefore, he underwent a closed thoracic drainage procedure on July 4, 2023. However, the ongoing air leakage did not improve. He underwent adjustments of the closed thoracic drainage along with continuous negative-pressure suction from June 13–28th 2023. He visited our hospital for follow-up examination on July 2, 2023. The main findings of the chest CT were as follows: 1. There was a pleural cavity encapsulated with air on the left, leading to approximately 40–50% compression of the left lung tissue, resulting incomplete pulmonary expansion. 2. Both lungs exhibited multiple infectious lesions and bronchiectasis, accompanied by interstitial changes. Notably, the left upper lobe showed partial destruction or absence, indicating the possibility of mycobacterial infection. Thoracoscopic surgery revealed a 20 × 15 cm cavity in the left lung, along with an irregular 2 × 3 cm lesion on the surface of the left upper lung on July 6, 2023. This lesion contained multiple necrotic tissue fragments, ranging from 0.5–1.0 cm, which were identified as Aspergillus nidulans. Accordingly, antifungal treatment, namely voriconazole (150 mg, q12 h), along with continuous chest irrigation was initiated on July 6, 2023. *S. marcescens* was initially detected in the hydrothorax on August 7, 2023. The bacterial load of *S. marcescens* during antibiotic treatment was monitored using a semi-quantitative method. Although several antibiotics, including clindamycin, cefuroxime, levofloxacin, and a combination of amikacin and tazocin, were administrated (Figure 1), *S. marcescens* infection was not ameliorated (Figure S1). Subsequent antibiotic susceptibility tests revealed intrinsic resistance of *S. marcescens* isolates to polymyxin B, ampicillin, and various cephalosporin antibiotics. Remarkably, treatment with levofloxacin promptly induced resistance to fluoroquinolones, including levofloxacin and ciprofloxacin (Table 1). Fungal cultures were negative after a 137-day treatment with voriconazole (Figure 1). Considering that the main suffering of this patient was a refractory infection with *S. marcescens*, an innovative therapeutic strategy combining phage treatment with antibiotics was planned.

**Table 1.** Minimum inhibitory concentrations (μg/mL) of selected antibiotics against S. marcescens **before** initiation of phage treatment.

Antibiotics		*Serratia marcescens* isolates (Days after hospital admission)								
		32	43	44	55	61	70	84	91	101
**β-lactams**	Ampicillin	> 16 (R)	> 16 (R)	ND	ND	> 16 (R)	> 16 (R)	> 16 (R)	> 16 (R)	> 16 (R)
	Amoxicillin	> 32 (R)	> 32 (R)	ND	> 32 (R)	> 32 (R)	> 32 (R)	> 32 (R)	> 32 (R)	> 32 (R)
**Aminoglycosides**	Gentamicin	≤ 2 (S)	≤ 2 (S)	≤ 2 (S)	≤ 2 (S)	≤ 2 (S)	≤ 2 (S)	≤ 2 (S)	≤ 2 (S)	≤ 2 (S)
	Tobramycin	4 (S)	4 (S)	ND	≤ 2 (S)	≤ 2 (S)	≤ 2 (S)	4 (S)	8 (I)	8 (I)
	Amikacin	≤ 8 (S)	≤ 8 (S)	ND	≤ 8 (S)	≤ 8 (S)	≤ 8 (S)	≤ 8 (S)	≤ 8 (S)	≤ 8 (S)
**Chloramphenicol**	Chloramphenicol	16 (I)	> 16 (R)	ND	> 16 (R)	> 16 (R)	> 16 (R)	> 16 (R)	> 16 (R)	> 16 (R)
**Tetracyclines**	Tetracycline	> 8 (R)	> 8 (R)	> 8 (R)	> 8 (R)	> 8 (R)	> 8 (R)	> 8 (R)	> 8 (R)	> 8 (R)
**Cephalosporins**	Cefoxitin	> 16 (R)	ND	ND	> 16 (R)	> 16 (R)	> 16 (R)	> 16 (R)	> 16 (R)	> 16 (R)
	Cefazolin	> 16 (R)	> 16 (R)	ND	> 16 (R)	> 16 (R)	> 16 (R)	> 16 (R)	> 16 (R)	> 16 (R)
	Cefuroxime	> 16 (R)	> 16 (R)	ND	> 16 (R)	> 16 (R)	> 16 (R)	> 16 (R)	> 16 (R)	> 16 (R)
	Ceftriaxone	≤ 1 (S)	≤ 1 (S)	ND	≤ 1 (S)	≤ 1 (S)	≤ 1 (S)	2 (I)	2 (I)	2 (I)
**Fluoroquinolones**	Levofloxacin	0.5 (S)	≤ 0.5 (S)	≤ 0.5 (S)	1 (I)	1 (I)	0.5 (S)	1 (I)	1 (I)	1 (I)
	Ciprofloxacin	0.25 (S)	≤ 0.25 (S)	≤ 0.25 (S)	1 (R)	0.5	0.25 (S)	1 (R)	1 (R)	1 (R)
**Carbapenems**	Imipenem	1 (S)	1 (S)	1 (S)	1 (S)	1 (S)	1 (S)	1 (S)	1 (S)	1 (S)
	Meropenem	≤ 0.125 (S)	≤ 0.125 (S)	ND	≤ 0.125 (S)	≤ 0.125 (S)	≤ 0.125 (S)	0.25 (S)	≤ 0.125 (S)	≤ 0.125 (S)
	Ertapenem	≤ 0.25 (S)	≤ 0.25 (S)	≤ 0.25 (S)	≤ 0.25 (S)	≤ 0.25 (S)	≤ 0.25 (S)	≤ 0.25 (S)	≤ 0.25 (S)	≤ 0.25 (S)
**Polypeptide**	Polymyxin B	ND	> 4 (R)	ND	> 4 (R)	ND	ND	ND	ND	ND

S: Susceptible, R: Resistant, I: Intermediate, ND: Not determined (ND).

### Selection of phage and verification of the synergic effects combining phage Spe5P4 and antibiotics

We isolated nine *S. marcescens* phages, among which Spe5P2 and Spe5P4 were selected for further analysis because of their clear plaques on agar plates and high plating efficiency. Subsequent genome sequencing and analysis revealed that Spe5P2 and Spe5P4 were the same phage, we then chose Spe5P4 for subsequent studies (Figure S2). Genome analysis indicated that phage Spe5P4 is free of virulence, lysogenic, and antibiotic-resistance genes (Figure S3A), suggesting its potential suitability for therapeutic purposes because of its safety [43]. Phylogenetic analysis of genomes of Spe54 (blue) and other 93 *Serratia* phages (black) in GenBank revealed that Spe5P4 belongs to the unnamed family 4 group (Figure S3B). Hence, Spe5P4 was selected as a candidate for phage treatment in this case. The characteristics of phage Spe5P4, including its morphological characteristics, sensitivity to temperature, pH, and MOI, are shown in Figure S2 (Figure S4). The thermal tolerance assay showed that phage Spe5P4 maintained stable infectivity after incubation at 25, 37, and 50 °C for 60 min (Figure S4B). The pH stability of Spe5P4 was tested across a range of pH values from 2–11. After 60 min of incubation, Spe5P4 exhibited stable infectivity within a broad pH range of 4–11 (Figure S4C). The optimal multiplicity of infection (MOI) for Spe5P4 was determined to be 0.1, at which point it produced the highest phage progeny, reaching approximately 1.01 × 10^10^ PFU/mL (Figure S4D). A phage-resistance induction assay for *S. marcescens* indicated a low resistance frequency at MOI values between 100 and 0.1 (3.33% to 6.66%), increasing to 10% at an MOI of 0.01 (Table S1).

The results of synergic effects assays showed that phage Spe5P4 achieved a satisfactory reduction in the bacterial population at a concentration of 10^9^ PFU/mL when co-treated with AMK (Figure 2B) and MEM (Figure 2C). The synergic effects of Spe5P4 and the antibiotics AMK and MEM were confirmed.

Finally, phage Spe5P4 was selected for the phage treatment for the following experiments.

### Verification of efficacy and safety of phage Spe5P4 treatment

#### Efficacy

**Figure 3.**
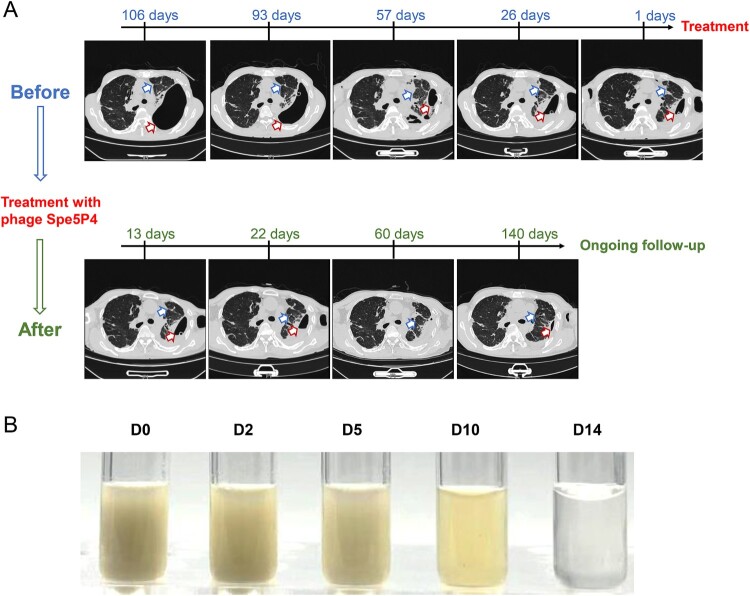
**Observation of the efficacy of the phage treatment with time.** (A) Representative chest CT images before (blue) and after phage treatment (green). Blue arrows present the lung tissue, red arrows present the pleural fluid. Before phage treatment, the lung tissue (black parts marked with a blue arrow) was markedly compressed indicating notable atelectasis. These black parts recovered slowly, indicating a slow recovery of atelectasis before phage administration. The pleural fluid (marked with a red arrow) was large and remarkable. The reduction in this part was slow, indicating slow absorption of the inflammatory hydrothorax. However, after phage treatment, the lung tussue achieved fast recovery, and the pleural fluid became smaller. At the end of follow-up, the lung tissue significantly recovered, and the pleural fluid was greatly aborted. Thus, phage treatment contributed to better amelioration of pulmonary conditions. (B) Appearance of the hydrothorax collected by thoracentesis. Obviously, the hydrothorax became clear with the phage treatment.

Importantly, the original complaints and symptoms of this patient markedly improved with the administration of phage Spe5P4. Figure 3 shows the amelioration of chest CT manifestations (Figure 3A) and the hydrothorax (Figure 3B). As shown as in Figure 3A, changes of chest CT images are displayed. Before phage Spe5P4 treatment, compression of the left lung tissue on the left lung was evident (blue arrows), and the absorption of pleural fluid was quite slow (red arrows). After treatment with phages, the left lung tissue markedly recovered. At the end of the follow-up, the lung tissue became clearer, and the pleural fluid was almost completely absorbed (Figure 3A). Figure 3B shows the changes in the hydrothorax corrected with thoracentesis. It can be confirmed that the hydrothorax became clearer with the phage treatment (Figure 3B). Thus, the efficacy of treatment with phage Spe5P4 was verified.

#### Safety

**Figure 4.**
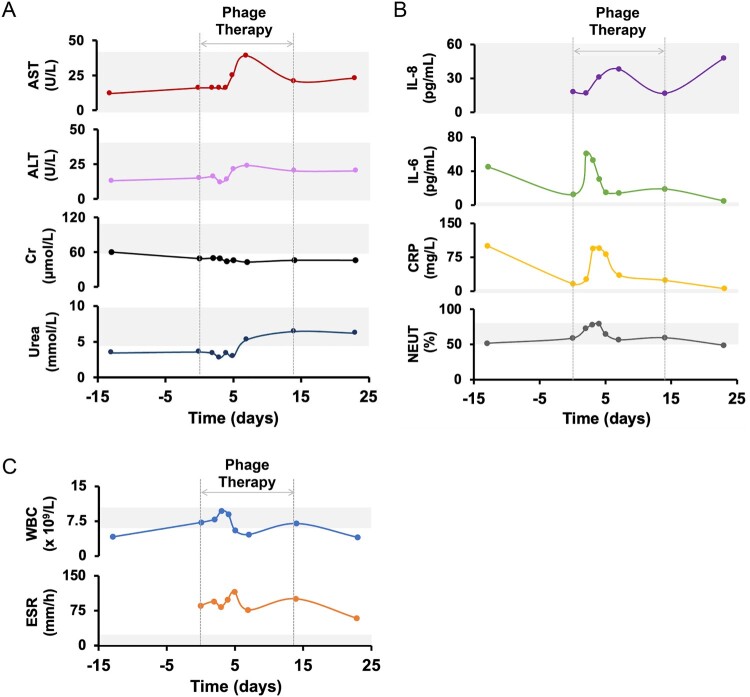
**Verification of the safety of phage treatment.** (A) Variations of the hepatic and renal functions influenced by phage treatment. Although there were mild fluctuations during the treatments, they almost came within the range of normal value (gray zone). (B) Variations of the immunity-related indices. Levels of IL-8, and NEUT changed within the range of normal value (gray zone). Levels of IL-6 and CRP were higher than normal value, but significantly reduced after treatment at 25-day follow-up. (C) Variations of the indices of blood routine examination. Levels of WBC and ESR were unchanged at 25-day follow-up, although there were fluctuations during the treatment. ESR were higher than the normal range, but it presents a reducing trend (amelioration). ALT: alanine aminotransferase, AST: aspartate aminotransferase, Cr: creatinine, CRP: C-reactive protein, ESR: erythrocyte sedimentation rate, IL-6,8: interleukin-6,8, NEUT%: neutrophil percentage, WBC: white blood cell.

Figure 4 shows the results of various laboratory examinations. In terms of the hepatic (AST, ALT) and renal (Cr, Urea) functions, the changes were analogous; although there were fluctuations during the treatment period, particularly during the phage administration period, all the changes fell into the range of normal values, and no significant difference was found before and after phage treatment (D-13 vs. D23) (Figure 4A). In terms of immunity-related indices, changes in IL-8 and NEUT (%) were analogous, namely, they changed within the range of normal values despite fluctuations during phage administration. Changes in IL-6 and CRP were analogous; namely, levels of IL-6 and CRP were higher than normal values, but after phage treatment, they were significantly ameliorated (Figure 4B). In terms of the indices of routine blood examination, WBC and ESR levels remained unchanged at the 25-day follow-up, although there were fluctuations during phage treatment. Changes in the WBC count were within the normal range. ESR levels were higher than the normal range, but unchanged before phage treatment (D0) and at the end of follow-up, although they presented an amelioration trend (reduced, Figure 4C).

Table 2 presents the results of the antibiotic susceptibility tests associated with phage Spe5P4 treatment. Susceptibility of most antibiotics was unchanged. Several antibiotics were prone to became susceptible with the phage treatment: Tobramycin (from 8 μg/mL–4 μg/mL, from intermediate to susceptible), Ceftriaxone (from 2 μg/mL– ≤ 1 μg/mL, from intermediate to susceptible), Levofloxacin (from 1 μg/mL–0.5 μg/mL, from resistant to susceptible), and Ciprofloxacin (from 1 μg/mL–0.5 μg/mL–0.25μg/mL, from resistant to intermediate to susceptible). Antibiotic resistance was not observed. Moreover, no phage-related adverse events occurred during the entire administration and follow-up periods.

**Table 2.** Minimum inhibitory concentrations (μg/mL) of selected antibiotics against Serratia marcescens **after** initiation of phage treatment.

Antibiotics		Serratia marcescens isolates (Days post phage therapy)				
		0	7	8	14	15
**β-lactams**	Ampicillin	> 16 (R)	> 16 (R)	ND	> 16 (R)	>16 (R)
	Amoxicillin	> 32 (R)	> 32 (R)	ND	> 32 (R)	> 32 (R)
**Aminoglycosides**	Gentamicin	≤ 2 (S)	≤ 2 (S)	≤ 2 (S)	≤ 2 (S)	≤ 2 (S)
	Tobramycin*	8 (I)	4 (S)	4 (S)	4 (S)	4 (S)
	Amikacin	≤ 8 (S)	≤ 8 (S)	≤ 8 (S)	≤ 8 (S)	≤ 8 (S)
**Chloramphenicol**	Chloramphenicol	> 16 (R)	> 16 (R)	ND	16 (I)	16 (I)
**Tetracyclines**	Tetracycline	> 8 (R)	> 8 (R)	ND	> 8 (R)	> 8 (R)
**Cephalosporins**	Cefoxitin	> 16 (R)	> 16 (R)	ND	> 16 (R)	> 16 (R)
	Cefazolin	> 16 (R)	> 16 (R)	ND	> 16 (R)	> 16 (R)
	Cefuroxime	> 16 (R)	> 16 (R)	ND	> 16 (R)	> 16 (R)
	Ceftriaxone*	2 (I)	≤ 1 (S)	ND	≤ 1 (S)	≤ 1 (S)
**Fluoroquinolones**	Levofloxacin*	1 (I)	1 (I)	0.5 (S)	0.5 (S)	0.5 (S)
	Ciprofloxacin*	1 (R)	0.5 (I)	0.25 (S)	0.25 (S)	0.25 (S)
**Carbapenems**	Imipenem	1 (S)	1 (S)	1 (S)	1 (S)	1 (S)
	Meropenem	≤ 0.125 (S)	≤ 0.125 (S)	ND	≤ 0.125 (S)	≤ 0.125 (S)
	Ertapenem	≤ 0.25 (S)	≤ 0.25 (S)	ND	≤ 0.25 (S)	≤ 0.25 (S)
**Polypeptide**	Polymyxin B	ND	ND	ND	ND	ND

S: Susceptible, R: Resistant, I: Intermediate, ND: Not determined (ND). * represents susceptibility to antibiotics ameliorated by phage treatment.

### Microbiological monitoring during phage therapy: verification of the phage-bacteria interactions

**Figure 5.**
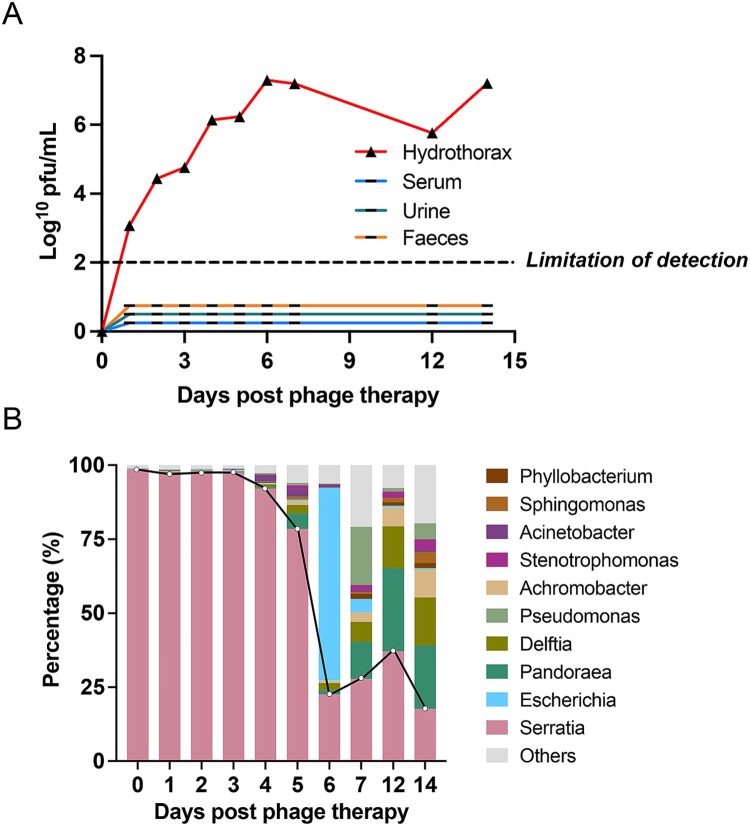
**Characteristics of microbiological interaction of phage Spe5P4 and bacteria during inpatient period.** (A) Distribution of phage Spe5P4 in hydrothorax, serum, urine and faeces samples during phage therapy. Only phage Spe5P4 in hydrothorax could be detected, whereas phage in serum, urine and faeces samples were under the limitation of detection. (B) Detection of the bacteria using a 16S rRNA analysis during period of inpatient phage treatment. *S. marcescens* (Serratia, pink column) was well displayed. It presented a notable and progressive reduction with the course of phage treatment.

Figure 5 shows the characteristics of the microbiological interaction between phage Spe5P4 and bacteria during the inpatient period. In terms of phage distribution, only phage Spe5P4 in hydrothorax samples could be detected, whereas phages in serum, urine, and fecal samples were under the limitation of detection. The phage concentration in the hydrothorax displayed a consistent upward trend, steadily escalating from the initial administration until day 6 and then maintained a consistently high level thereafter. This result suggests that the hydrothorax samples are sensitive to the detection of phage Spe5P4 concentrations (Figure 5A). The abundance of *S. marcescens* in the hydrothorax samples during phage antibiotic treatment was also checked using 16S rRNA analysis. The results showed a notable and progressive decrease in *S. marcescens* load over the treatment period (Figure 5B), which provided further evidence of the therapeutic effects of phage Spe5P4 treatment against *S. marcescens.*

**Figure 6.**
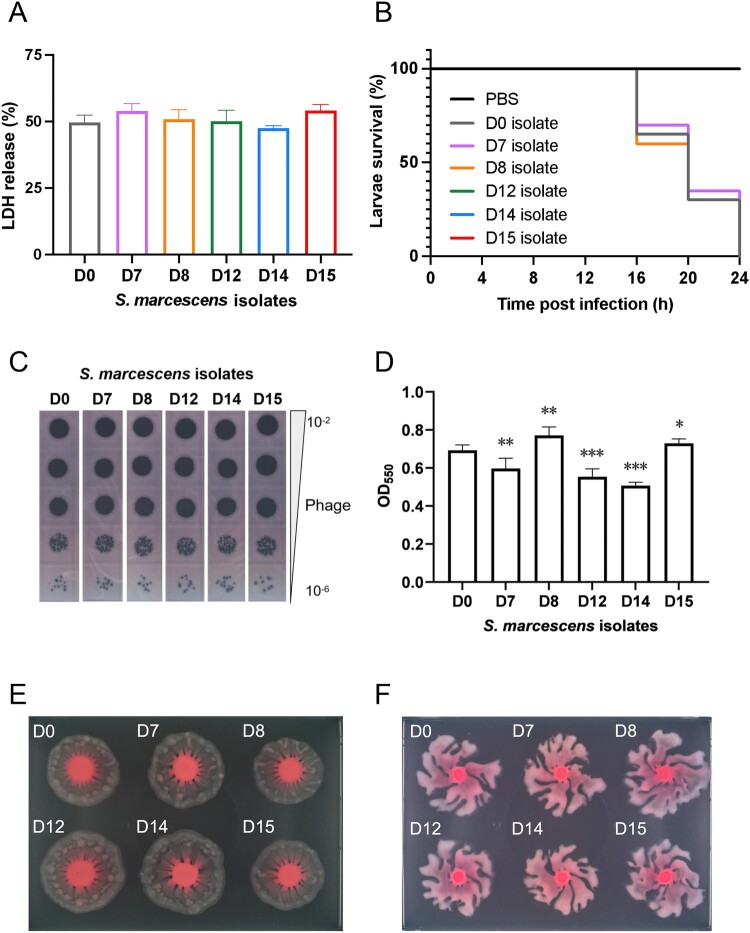
**Investigation of the potential phage-bacteria interactions between phage Spe5P4 and *S. marcescens.*** (A) LDH release of the *S. marcescens* on A549 cells. The LDH release was unchanged with time of phage treatment. (B) Kaplan-Meier plots of the virulence in *S. marcescens* evaluated using a *G. mellonella* infection model. The Kaplan – Meier plots represent the survival of 20 larvae that were used for each strain. (C) Visualization of plaque forming units of the *S. marcescens* on the patient’s strains isolated before (D0) and during phage treatment (D7 – D15). (D) Value of biofilm formation capacity (OD_50_) of the *S. marcescens* before and during phage treatment. (E) Examination of the swarming capacity of the *S. marcescens* before and during phage treatment. (F) Examination of the swimming capacity assay of the *S. marcescens* before and during phage treatment. * represents *p* < 0.05, ** represents *p* < 0.01, *** represents *p* < 0.001 (vs. the D0 isolate).

Figure 6 shows that the cytotoxicity and virulence of *S. marcescens* were influenced by phage treatment in A549 cells. We found that with phage treatment (From D0–D15), no significant changes in LHD release were detected (vs. D0, Figure 6A). In terms of the results of the *G. mellonella* infection assay, the Kaplan-Meier curve of the virulence in *S. marcescens* was almost unaffected by phage treatment (Figure 6B), which was consistent with the data of LHD release. These data confirmed that phage exposure did not change the virulence of *S. marcescens*. Phenotypic heterogeneity of *S. marcescens* affected by phage treatment. The results of EOP experiments demonstrated that phage-resistant bacterial strains did not emerge during phage treatment (Figure 6C). Whole genome sequence analysis of *S. marcescen* isolates during phage therapy indicated no phage resistance related gene mutation (Table S2). The biofilm formation capacity (OD_50_) of *S. marcescens* before and during phage treatment indicated a noteworthy reduction in the biofilm-forming abilities of D7, D12, and D14. However, the biofilm-forming capacity increased on D8 and D15 (Figure 6D). The swarming capacity (Figure 6E) and swimming capacity (Figure 6F) exhibited an inverse trend to biofilm formation. The dynamic changes in *S. marcescens* caused by phage treatment are shown (Figure 6).

Phage treatment significantly reduced the bacterial load of *S. marcescens.* However, it did not increase the cytotoxicity or virulence of *S. marcescens*. No phage-resistant bacterial strains emerged during the phage-treatment period. In terms of the biofilm-forming, swarming, and swimming capacities of *S. marcescens,* dynamic changes were observed with phage treatment.

## Discussion

Infection with multidrug-resistant strains of *S. marcescens* has been a serious problem in the clinical setting since the administration of antibiotics alone cannot achieve satisfactory efficacy. Here, by collaborating with a patient with pulmonary infection of *S. marcescens,* we completed the present study to verify the feasibility of applying phage treatment against refractory *S. marcescens* infection. Through our efforts, we selected the appropriate phage, Spe5P4, against *S. marcescens* infection. We also verified the efficacy and safety of phage treatment. Finally, we investigated the phage-bacteria interactions that are indispensable for the better application of phage Spe5P4 in the future. To the best of our knowledge, this is the first report elucidating the application of phage treatment in treating refractory infections caused by multidrug-resistant *S. marcescens*. The findings of this report are helpful for the exploration of novel treatments against refractory *S. marcescens* infections. In addition, we provided a methodological basis for the clinical application of phage treatment against refractory clinical infections, since phage-based therapy should be a future direction against refractory bacterial infections, independent of the development of novel antibiotics.

### Regarding the patient and clinical setting

Patients with pulmonary infections were recruited for this study. *S. marcescens* was detected in the hydrothorax. Although he underwent treatment with many antibiotics (Figure 1), the *S. marcescens* infection was not ameliorated. The results of the first antibiotic susceptibility test indicated that he was suffering from a multidrug-resistant *S. marcescens* infection (Table 1). This patient was an ideal subject to investigate the therapeutic effects of phages against *S. marcescens*, and was therefore considered to be submitted for phage treatment.

In terms of the therapeutic protocol, we considered to use the plan of “antibiotics + phage treatment” rather than sole application of phage because this is the first study using phage treatment, of which the phage-related efficacy was uncertain. Sole administration of antibiotics without antibiotic treatment is risky. Accordingly, efficacy was evaluated by evaluating the synergic effects of antibiotics and phage treatment. Two antibiotics, SMK and MEM, were selected as antibiotics combined with phage treatment after comprehensively considering their susceptibility and safety.

### Phage Spe5P4 is an ideal phage against *S. marcescens* infection

First, our genome analysis results indicated that phage Spe5P4 was not associated with virulence, lysogenic, or antibiotic-resistance genes (Figure S1A). Second, the *in vitro* tests for the synergic effects of phage Spe5P4+ AMK (Figure 2B) and phage Spe5P4+ MEM (Figure 2C) suggested satisfactory synergistic effects of the combination of phage treatment and antibiotics. Accordingly, Phage Spe5P4 is appropriate for treating *S. marcescens* infections. In this study, only Spe5P4 was used for therapy, while the use of single phages in phage therapy carries a higher risk of phage-resistant bacteria emergence, and phage cocktails provide a more robust approach by leveraging the diversity and synergy of multiple phages. This reduces the selective pressure on bacterial populations, enhances therapeutic efficacy, and lowers the probability of treatment failure due to resistance. It is important to note that phage resistance can develop in both single-phage and cocktail treatments [44, 45], and a potential strategy to mitigate the emergence of resistant strains involves the use of pre-optimized phages [16, 46–48]. Throughout the treatment process, we actively isolated virulent phages to ensure that new phages could be utilized in the development of phage resistance. Phage susceptibility testing of the *S. marcescens* isolates demonstrated no evidence of resistance. Future phage therapy protocols should prioritize the use of pre-optimized cocktails to maximize their effectiveness and minimize the emergence of resistant bacterial strains.

### Phage treatment exhibited satisfactory efficacy and safety

The marked amelioration of clinical manifestations in this patient suggested the efficacy of phage treatment. In addition to alleviation of symptoms, the chest CT images (Figure 3A) along with the hydrothorax examinations (Figure 3B) gradually recovered with the administration of the phage. Although we could not set a control group here, comparison of the recovery situation before and after phage treatment may confirm the efficacy of phage-related treatment.

With respect to safety, administration of phage did not change hepatic and renal functions (Figure 4A), IL-8 and NEUT (%) (Figure 4B), and WBC and ESR levels (Figure 4C). The levels of IL-6 and CRP were prone to amelioration (Figure 4C). Based on semi-quantitative bacterial analysis and evaluation of *S. marcescens* abundance in hydrothorax samples, bacterial load and abundance significantly decreased following phage treatment. Bacterial lysis by phages results in the release of a large quantity of lipopolysaccharides (LPS), which subsequently stimulates the patient's immune response. This immune activation leads to elevated levels of IL-6 and CRP. However, after seven days of treatment, both IL-6 and CRP levels began to decrease and ultimately returned to normal by the 21st day. Our data from the second antibiotic susceptibility test indicated that phage treatment did not enhance resistance to all the antibiotics involved. Conversely, several antibiotics were susceptible to phage treatment (Table 2). Phage treatment neither deteriorated the results of laboratory examinations nor enhanced the drug resistance to all antibiotics. Importantly, no age-related adverse events were observed during the protocol. Hence, phage treatment had satisfactory safety.

These results indicated that phage treatment markedly ameliorated the clinical manifestations of this patient but seldom influenced the hepatic and renal functions, immunity-related indices, and indices of routine blood examinations (Figure 4). Moreover, resistance to antibiotics was not reduced by phage treatment (Table 2). Accordingly, the efficacy and safety of the phage treatment were verified.

However, in order to achieve rigorous verification of the efficacy of antibiotics combined with phage administration in the treatment process, studies such as exploration of the optimal antibiotic dosage, time interval and mode of administration, comparison of the treatment results with antibiotic therapy alone, and the design of a controlled trial are extremely important. However, this study performed an explorative observation in a clinically difficult case. Thus, the situation of this patient did not allow us to perform such rigorous verifications. These studies will be planned in the future investigation.

### Phage Spe5P4 has satisfactory phage-bacteria interactions

One concern with phage treatment is that it may increase the cytotoxicity and virulence of the targeted bacterium [49–51]. Meanwhile, phage-resistant bacterial strains may emerge with phage treatment [52, 53]. For better application of phages, the evaluation of the interactions between phage SPe5P4 and *S. marcescens* is indispensable.

Our results indicate that phage treatment significantly reduced the bacterial load of *S. marcescens* (Figure 5)*.* However, it did not increase the cytotoxicity (Figure 6A) or virulence (Figure 6B) of *S. marcescens*. No phage-resistant bacterial strains emerged during the phage-treatment period (Figure 6C). The capacity for biofilm formation (Figure 6C, D), swarming (Figure 6E), and swimming (Figure 6F) of *S. marcescens,* it exhibited dynamic changes with phage treatment, and the pathophysiological significance required further investigation.

Overall, phage Spe5P4 did not increase the bacterial load, cytotoxicity, virulence, or resistance of *S. marcescens.* It can be employed for future treatment and research on *S. marcescens* infections.

### Strengths, limitations and future perspectives

This study has several strengths. *i*) This is the first study to verify the application of phage treatment in treating refractory infections of multidrug-resistant *S. marcescens*. We selected an appropriate patient with good compliance, along with an appropriate phage Spe5P4, which enabled the implementation of the present study. *ii*) The efficacy, safety, and phage-bacteria interactions of phage Spe5P4 were verified, which provide evidence and working basis for further investigation of using phage Spe5P4 in treating refractory *S. marcescens* infection. *iii*) We provide a methodological basis for future development and research on the clinical application of phage treatment against refractory bacterial infections.

This study also suffers from several limitations, since this is a pioneering study using phages to treat *S. marcescens* infection: *i*) Only data from a single patient were collected and analyzed. We could not establish a research cohort conducting a case–control study, which might have resulted in biased results and weakened the strength of the evidence. *ii*) No previous analogous studies were used for comparison.

Based on the evidence, methodology, and insights obtained in the present study, future studies should focus on the following issues: *i*) Sole administration of phage Spe5P4 could be considered. *ii*) The establishment of a cohort that includes patients with refractory *S. marcescens* infection is indispensable. *iii)* Owing to the explorative nature of this study, we did not perform a complete assessment of safety and after-effects by documenting the discontinuation of treatment after symptomatic improvement and adverse effects at post-discharge follow-up. These issues should be addressed in future research. *iv*) To verify the safety and efficacy of phage Spe5P4, using an experimental design such as sole phage treatment vs. antibiotics vs. phage treatment ± antibiotics might be more reasonable to obtain more rigorous evidence.

Recently reported phage lysins exhibit efficient bactericidal activity for *Escherichia coli* O157:H7, *Klebsiella pneumoniae, Pseudomonas aeruginosa*, and Other Gram-Negative ESKAPE Pathogens [54, 55]. In the future, phage lysins may also be considered as a promising alternative for antimicrobial therapy. For example, DeepLysin is the first artificial intelligence (AI)-based platform for discovering antimicrobial proteins, capable of identifying highly efficient lysins from diverse “dark matter” sources like phages and metagenomes. It uses AI to efficiently screen and pinpoint subtle, high-dimensional features of potent antimicrobial proteins. The lysin candidate LLysSA9 identified through DeepLysin exhibits best-in-class activity and shows promising therapeutic effects in animal models of systemic infection. This indicates that DeepLysin has broad application prospects in the prevention and control of clinical epidemic pathogens, particularly ESKAPE pathogens. Utilizing DeepLysin to discover high-activity antimicrobial protein candidates can effectively combat infections caused by drug-resistant bacteria, playing a significant role in the prevention and control of zoonotic diseases caused by drug-resistant bacteria [56, 57].

## Conclusions

By collaboration with a proper patient with refractory pulmonary infection by multidrug-resistant *S. marcescens,* we conducted the present study to explore the feasibility of using phage in treating multidrug-resistant *S. marcescens* infection. First, we confirmed that Spe5P4 is an ideal phage against *S. marcescens* infection. Subsequently, the efficacy and safety of Spe5P4 were verified. Phage – bacteria interactions between phage Spe5P4 and *S. marcescens* were explored. The findings of the present study provide evidence and a working basis for further investigation of phage Spe5P4 in the treatment of refractory *S. marcescens* infection. We also provided a methodological basis for future development and research on the clinical application of phage treatment against refractory bacterial infections. However, because of the explorative nature of this study, many rigorous verifications could not be conducted owing to the clinical situation of this patient, which restricted us from obtaining more robust evidence. For better verification the safety/efficacy of the phage treatment, several considerations are indispensable: *i*) in the phage domain, standard procedures for selection and production of phage should be established; *ii*) in the combined antibiotics domain, selection of appropriate antibiotics also requires a standard operating procedure; *iii*) many technical details should be addressed, such as development of the phage treatment-specific assessments, establishment of quality control standards for phage production, as well as establishment of the phage-related risk prevention system; *iv*) well-designed, large-scale, and multi-centre randomized controlled trials are anticipated to get the robust evidence. These issues need to be well considered for the future research and development of phage treatment as a sharp sword against multi-drug resistant bacteria.

## Supplementary Material

Figure S1.tif

Supplementary Material MSRV1.docx

Figure S4.tif

Figure S2.tif

Table S1.docx

Table S2.docx

Figure S3.tif

## Data Availability

The phage sequence has been submitted to GenBank under the accession number # PP858852.
