# Supplementary figures and images for: Exploration of the feasibility of clinical application of phage treatment for multidrug-resistant Serratia marcescens-induced pulmonary infection
Source: Emerg Microbes Infect. 2025 Jan 7;14(1):2451048. doi: 10.1080/22221751.2025.2451048 (PMC11740298; doi:10.1080/22221751.2025.2451048)

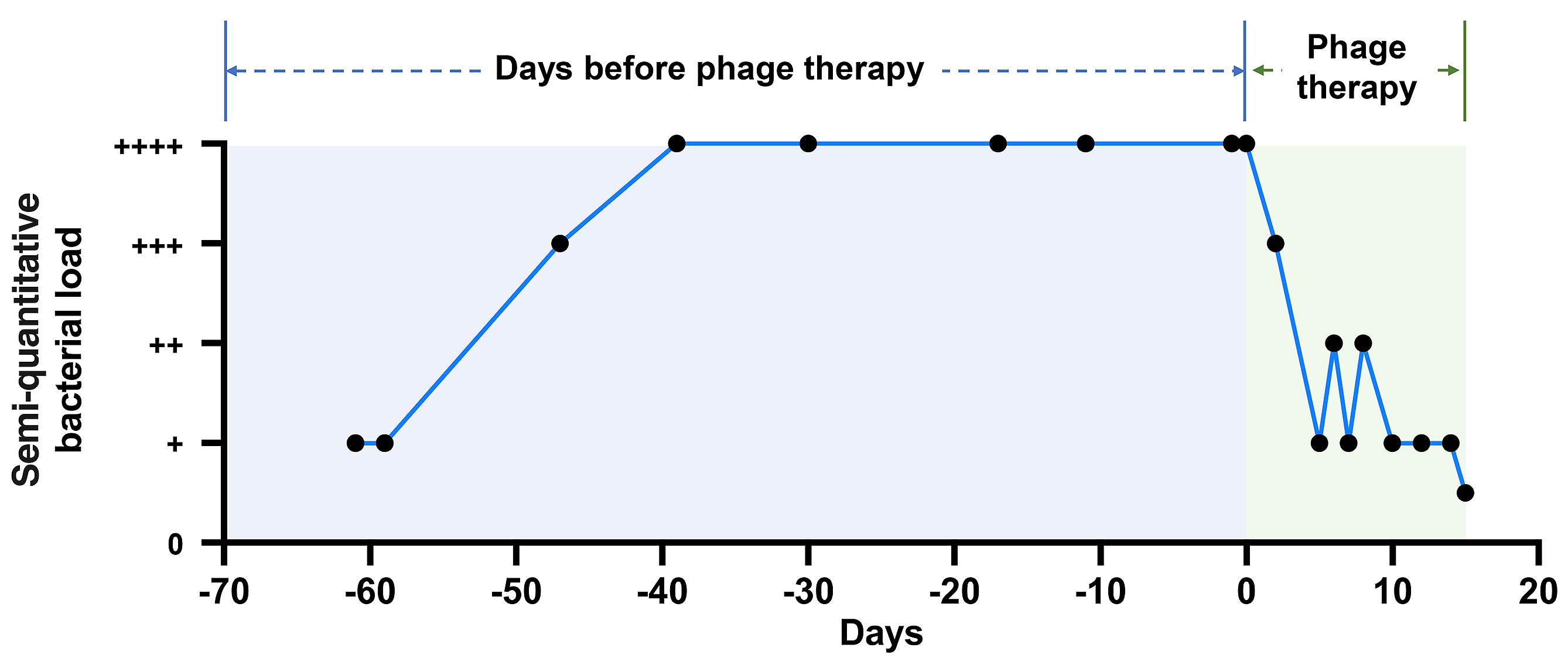

Supplement: Figure S1.tif [file TEMI_A_2451048_SM9289.tif]

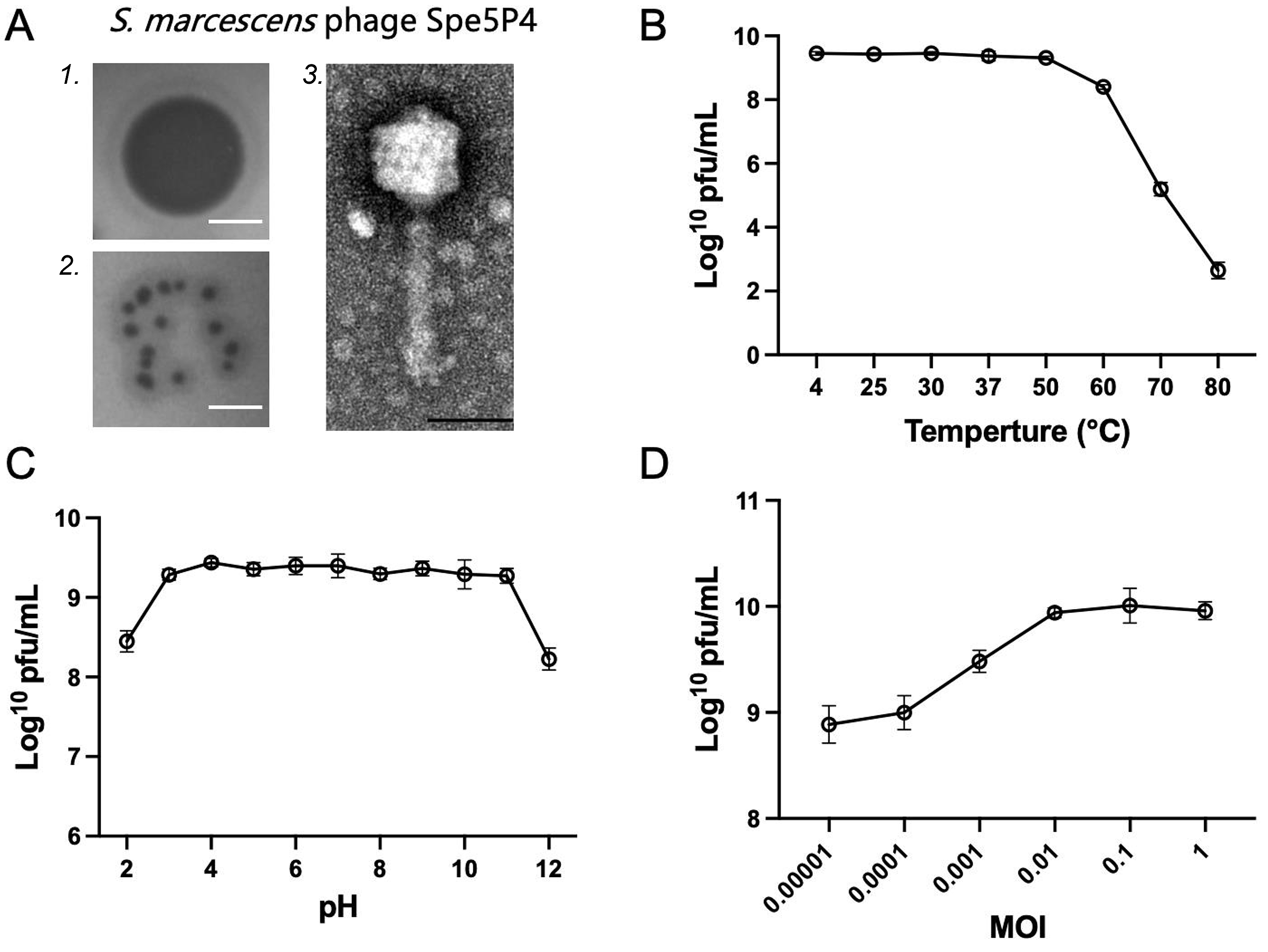

Supplement: Figure S4.tif [file TEMI_A_2451048_SM9287.tif]

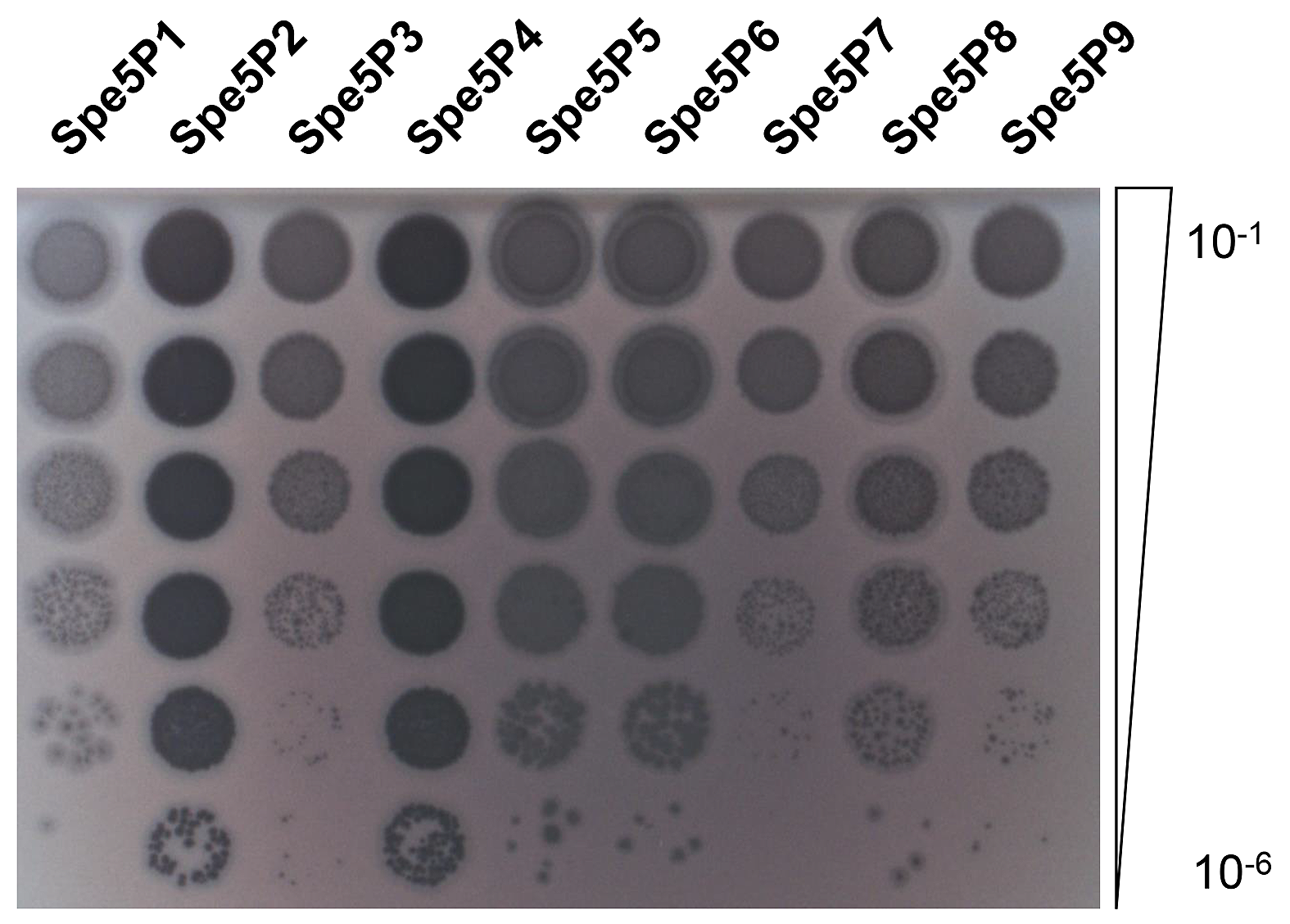

Supplement: Figure S2.tif [file TEMI_A_2451048_SM9286.tif]

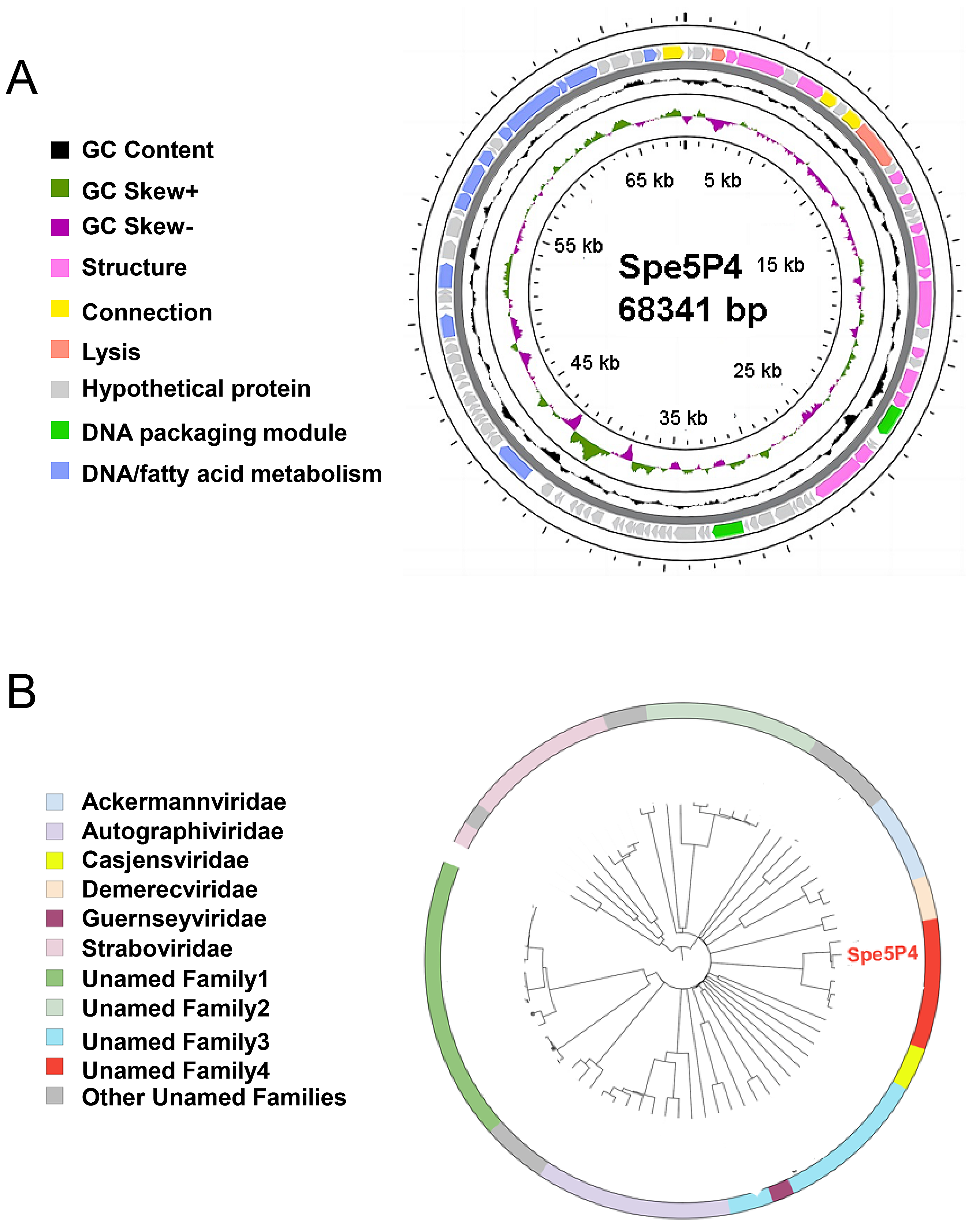

Supplement: Figure S3.tif [file TEMI_A_2451048_SM9283.tif]
