# Supplementary material for: Exploration of the feasibility of clinical application of phage treatment for multidrug-resistant Serratia marcescens-induced pulmonary infection
Source: Emerg Microbes Infect. 2025 Jan 7;14(1):2451048. doi: 10.1080/22221751.2025.2451048 (PMC11740298; doi:10.1080/22221751.2025.2451048)
Supplement: Supplementary Material MSRV1.docx [file TEMI_A_2451048_SM9288.docx]

**Supplementary materials**

**Table S1.** The phage-resistant frequency of *S. marcescens*

| MOI | 100 | 10 | 1 | 0.1 | 0.01 |
| --- | --- | --- | --- | --- | --- |
| Frequency | 3.33% (1/30) | 3.33% (1/30) | 6.66% (2/30) | 3.33% (1/30) | 10% (3/30) |

**Table S2.** List of chromosomal mutations identiﬁed in the genome of the *S. marcescen* isolates during phage therapy compare to *S. marcescens* 328505.

| **Isolates** | **Genes** | **Products** | **Mutations** | **Effects on the protein** | **Impact on the protein function** |
| --- | --- | --- | --- | --- | --- |
| **D7** | *cysJ* | Sulfite reductase flavoprotein alpha-component | 20C>T | synonymous variant | Unlikely |
| **D8** | *cysJ* | Sulfite reductase flavoprotein alpha-component | 20C>T | synonymous variant | Unlikely |
| **D12** | *cysJ* | Sulfite reductase flavoprotein alpha-component | 20C>T | synonymous variant | Unlikely |
|  | *pgaA* | Poly-beta-1,6-N-acetyl-D-glucosamine export protein | 20A>G | synonymous variant | Unlikely |
| **D14** | *cysJ* | Sulfite reductase flavoprotein alpha-component | 20C>T | synonymous variant | Unlikely |
| **D15** | *cysJ* | Sulfite reductase flavoprotein alpha-component | 20C>T | synonymous variant | Unlikely |

**Figure S1.** Examination of *S. marcescens* load by semi-quantitative method during the treatment.


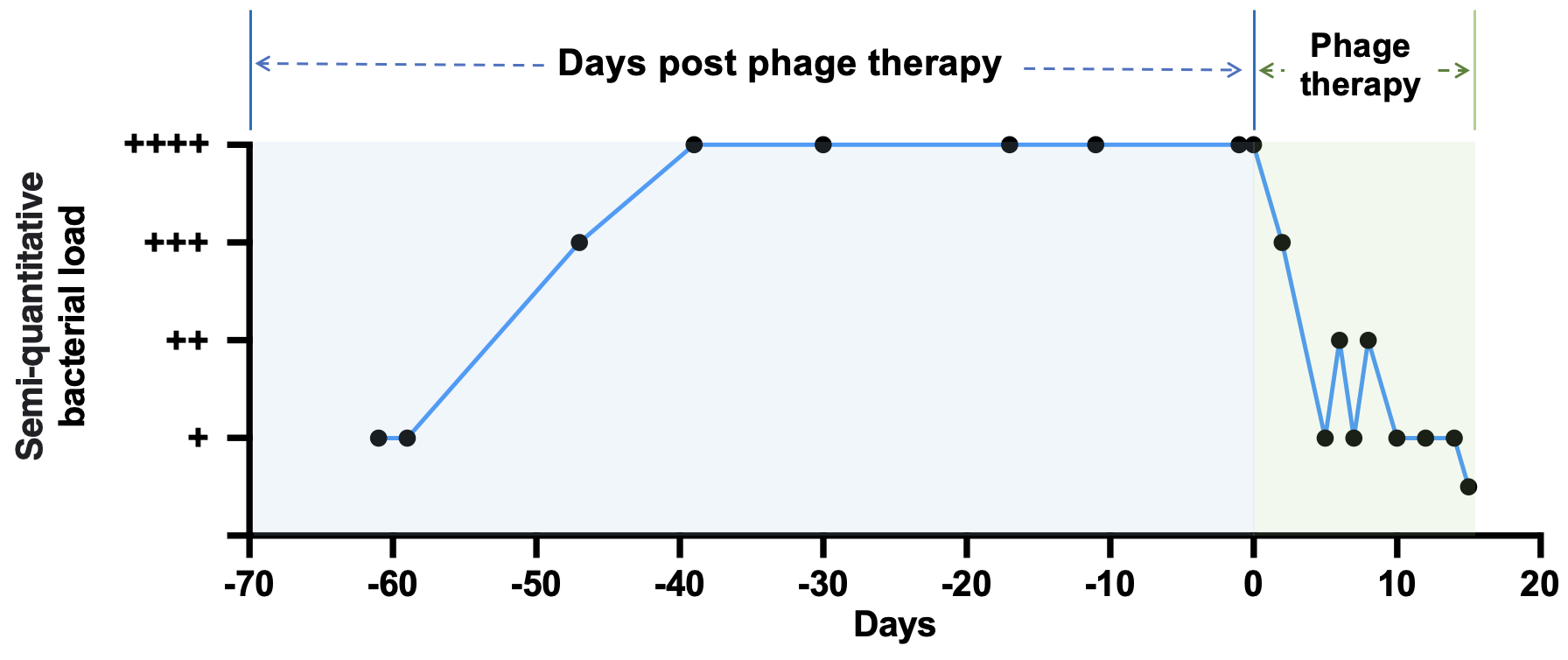


**Figure S2.** Efficiency of plating (EOP) of Serratia phages against *S. marcescens* 328505


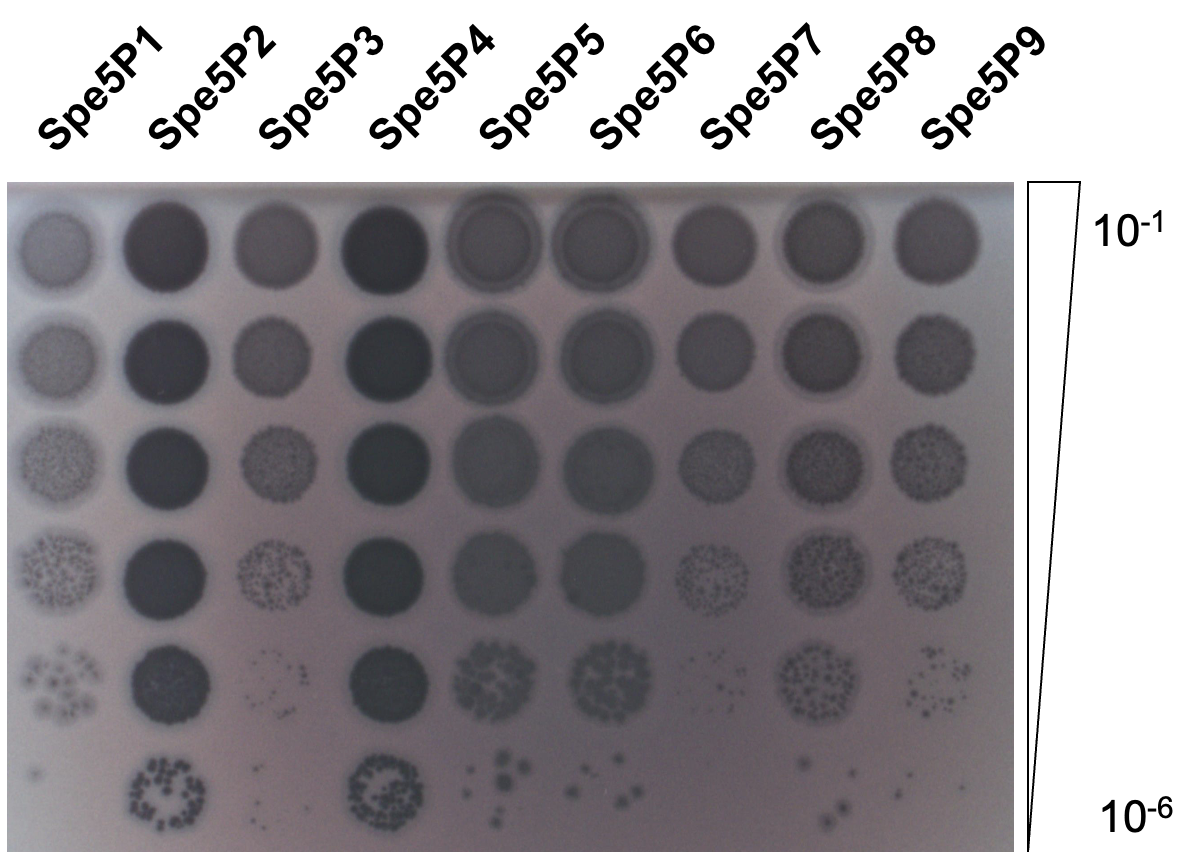


**Figure S3.** Genome analysis and phylogenetic tree of phage Spe5P4.


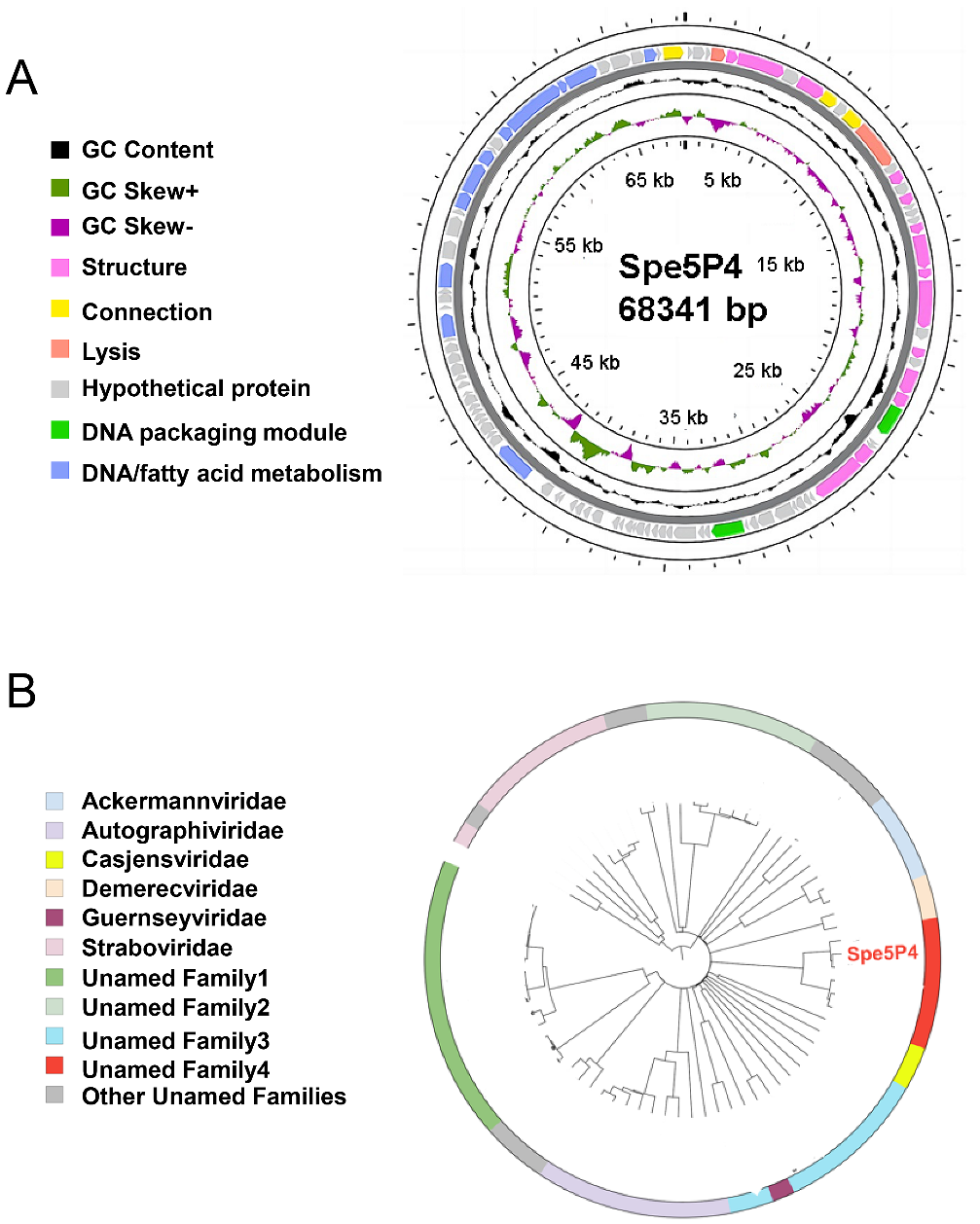


1. Genome analysis of phage Spe5P4.
2. Phylogenetic tree of phage Spe5P4.

**Figure S4.** Confirmation of the Characteristics of phage Spe5P4.


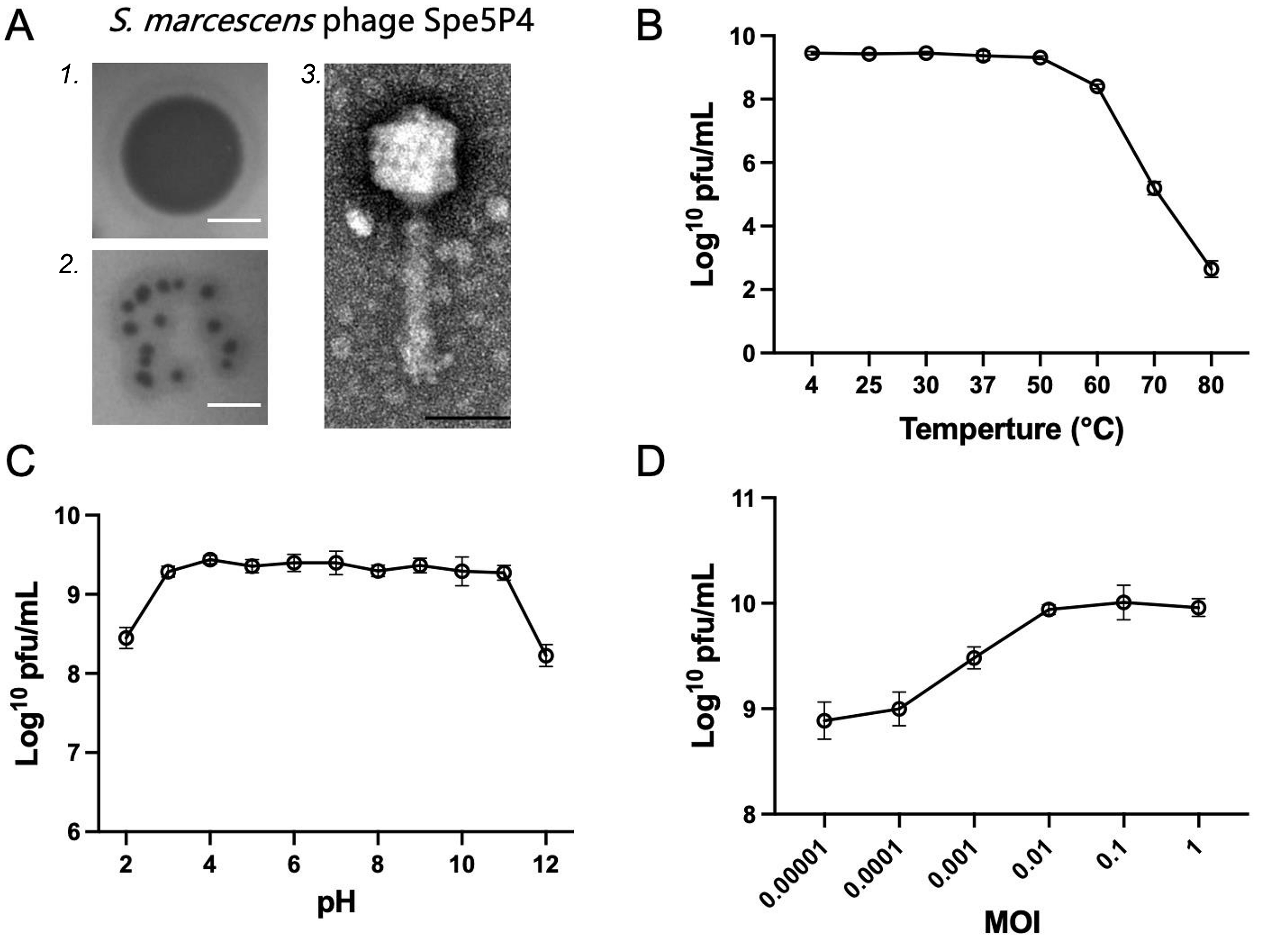


1. Representative electron microscopy images of phage Spe5P4. Plague morphology: 1 (bar = 5 mm) and 2 (bar =5 mm); transmission:3 (bar = 100 nm).
2. Verification of the temperature stability of phage Spe5P4.
3. Verification of the pH stability of phage Spe5P4.
4. Optimal multiplicity of infection (MOI) of phage Spe5P4.
