# Supplementary material for: Exploration of the feasibility of clinical application of phage treatment for multidrug-resistant Serratia marcescens-induced pulmonary infection
Source: Emerg Microbes Infect. 2025 Jan 7;14(1):2451048. doi: 10.1080/22221751.2025.2451048 (PMC11740298; doi:10.1080/22221751.2025.2451048)
Supplement: Table S1.docx [file TEMI_A_2451048_SM9285.docx]

**Table S1.** The phage-resistant frequency of *S. marcescens*

| MOI | 100 | 10 | 1 | 0.1 | 0.01 |
| --- | --- | --- | --- | --- | --- |
| Frequency | 3.33% (1/30) | 3.33% (1/30) | 6.66% (2/30) | 3.33% (1/30) | 10% (3/30) |
