# Supplementary material for: Exploration of the feasibility of clinical application of phage treatment for multidrug-resistant Serratia marcescens-induced pulmonary infection
Source: Emerg Microbes Infect. 2025 Jan 7;14(1):2451048. doi: 10.1080/22221751.2025.2451048 (PMC11740298; doi:10.1080/22221751.2025.2451048)
Supplement: Table S2.docx [file TEMI_A_2451048_SM9284.docx]

**Table S2.** List of chromosomal mutations identiﬁed in the genome of the *S. marcescen* isolates during phage therapy compare to *S. marcescens* 328505.

| **Isolates** | **Genes** | **Products** | **Mutations** | **Effects on the protein** | **Impact on the protein function** |
| --- | --- | --- | --- | --- | --- |
| **D7** | *cysJ* | Sulfite reductase flavoprotein alpha-component | 20C>T | synonymous variant | Unlikely |
| **D8** | *cysJ* | Sulfite reductase flavoprotein alpha-component | 20C>T | synonymous variant | Unlikely |
| **D12** | *cysJ* | Sulfite reductase flavoprotein alpha-component | 20C>T | synonymous variant | Unlikely |
|  | *pgaA* | Poly-beta-1,6-N-acetyl-D-glucosamine export protein | 20A>G | synonymous variant | Unlikely |
| **D14** | *cysJ* | Sulfite reductase flavoprotein alpha-component | 20C>T | synonymous variant | Unlikely |
| **D15** | *cysJ* | Sulfite reductase flavoprotein alpha-component | 20C>T | synonymous variant | Unlikely |
